# Phase I study of TAS-102 and irinotecan combination therapy in Japanese patients with advanced colorectal cancer

**DOI:** 10.1007/s10637-015-0271-1

**Published:** 2015-07-12

**Authors:** Toshihiko Doi, Takayuki Yoshino, Nozomu Fuse, Narikazu Boku, Kentaro Yamazaki, Wasaburo Koizumi, Ken Shimada, Yasutaka Takinishi, Atsushi Ohtsu

**Affiliations:** Department of Gastroenterology and Gastrointestinal Oncology, National Cancer Center Hospital East, 6-5-1 Kashiwanoha, Kashiwa, Chiba 277-8577 Japan; Division of Gastrointestinal Oncology, Shizuoka Cancer Center, 1007 Shimonagakubo, Nagaizumi, Sunto-gun, Shizuoka, 411-8777 Japan; Department of Gastroenterology, Kitasato University School of Medicine, 2-1-1 Asamizodai, Minami-ku, Sagamihara, Kanagawa 252-0380 Japan; Department of Internal Medicine, Showa University Yokohama Northern Hospital, 35-1 Chigasakityuo, Tsuzuki-ku, Yokohama, Kanagawa 224-8503 Japan; Exploratory Oncology Research & Clinical Trial Center, National Cancer Center, 6-5-1 Kashiwanoha, Kashiwa, Chiba 277-8577 Japan; National Cancer Center Hospital, 5-1-1 Tsukiji, Chuo-ku, Tokyo 216-8511 Japan; Showa University Koto Toyosu Hospital, 5-1-38 Toyosu, Koto-ku, Tokyo 135-8577 Japan

**Keywords:** TAS-102, Irinotecan, Phase I combination study, FTD, TPI, Colorectal cancer

## Abstract

*Background TAS*-102 is a nucleoside antitumor agent consisting of trifluridine (FTD) and tipiracil hydrochloride (TPI). We investigated the recommended dose (RD) of TAS-102 plus irinotecan for metastatic colorectal cancer refractory to 5-fluorouracil (5-FU) and oxaliplatin. *Methods* This study was used a escalated dose of TAS-102 (40–70 mg/m^2^/day, for 5 days a week with 2 days rest for 2 weeks, followed by a 14-day rest) with a fixed dose of irinotecan (150 mg/m^2^ on Days 1 and 15 of a 28-day schedule). The primary endpoints were determination of RD and assessment of safety. *Results* Ten patients were enrolled; 7 at the Level 1 (50 mg/m^2^/day) and 3 at the Level 2 (60 mg/m^2^/day). One patient at Level 1 was excluded from the analysis of dose-limiting toxicities (DLT) and efficacy. Five DLTs occurred in 3 patients; 1 patient at Level 1 (Grade 3 febrile neutropenia and Grade 4 neutropenia), and 2 patients at Level 2 (Grade 3 febrile neutropenia in two patients and Grade 4 neutropenia in one). Grade 3 or higher treatment-related adverse events were neutropenia (100 %), leukopenia (70 %), febrile neutropenia (30 %) and lymphopenia, anaemia (20 % each). 2 patients (22 %) achieved partial response with the duration of response were 112 and 799 days. *Conclusion* The RD was determined to be 50 mg/m^2^/day of TAS-102 combined with 150 mg/m^2^ of irinotecan although further investigation to explore optimal regimen is warranted.

## Introduction

TAS-102 (Taiho Pharmaceutical, Tokyo, Japan) is an oral antitumor agent, consisting of trifluridine (FTD; α,α,α-trifluorothymidine) and tipiracil hydrochloride [TPI; thymidine phosphorylase inhibitor; 5-chloro-6-(2-iminopyrrolidin-1-yl) methyl-2,4(1*H*,3*H*)-pyrimidinedione hydrochloride] at a molar ratio of 1:0.5. FTD is the active antitumor component of TAS-102, and its triphosphate form is incorporated into DNA in tumor cells [1, 2]. The incorporation into DNA is thought to contribute to antitumor effect of FTD. TPI is a potent inhibitor of thymidine phosphorylase that degrades FTD [3]. This mechanism of action is different from other cytotoxic and targeted agents, and TAS-102 was expected to be effective against various tumors resistant to other drugs.

A large body of clinical safety and efficacy data has been collected for TAS-102 [4–7]. These studies established a treatment schedule for TAS-102 monotherapy which consisted of twice-daily oral administration of TAS-102 on Days 1–5 and Days 8–12 in a 28-day treatment cycle. In a Japanese phase I clinical study, 70 mg/m^2^/day was determined as the RD of TAS-102 monotherapy in patients with solid tumors refractory to standard chemotherapy [8]. For metastatic colorectal cancer, TAS-102 monotherapy showed a survival benefit over best supportive care after standard chemotherapy failure [9]. While TAS-102 monotherapy is promising for metastatic colorectal cancer, it is warranted to enhance its antitumor efficacy by combination with other agents especially for earlier treatment lines for metastatic colorectal cancer.

In a preclinical study, combination of TAS-102 and several cytotoxic agents demonstrated synergistic effects, and the antitumor effect of combination of TAS-102 with irinotecan seemed the most promising (unpublished data). It was reported that SN-38, an active metabolite of irinotecan, induces DNA strand breaks and G2/M arrest is increased in combination with FTD [10]. Other studies showed that TAS-102 is also effective against human tumor cell lines which acquired resistance to 5-FU [11]. Therefore, the combination with TAS-102 and irinotecan is considered to be a new candidate for metastatic colorectal cancer refractory to initial therapy with 5-FU-based chemotherapy.

The primary objective of this phase I study was to determine the RD of the combination of TAS-102 plus irinotecan for future clinical trials in patients with metastatic colorectal cancer refractory to both fluoropyrimidine and oxaliplatin, and to evaluate the safety. Secondary objectives included the assessment of antitumor efficacy and pharmacokinetic (PK) interaction in this combination treatment regimen. In addition, this study explored the impact of UGT1A1 on toxicity and efficacy including its relation to *KRAS* status.

## Patients and methods

### Patient eligibility

Key inclusion criteria were as follows: (1) Japanese patients with histologically confirmed unresectable metastatic or recurrent cancer of the colon or rectum, and confirmed progressive disease in one prior chemotherapy containing a fluoropyrimidine and oxaliplatin; (2) At least one measurable lesion according to the Response Evaluation Criteria in Solid Tumors (RECIST) version 1.0; (3) No history of treatment with irinotecan; (4) Age from 20 to 74 years; (5) Eastern Cooperative Oncology Group Performance Status (PS) of 0 or 1; (6) Adequate bone marrow function (platelet counts ≥100,000/mm^3^, haemoglobin levels ≥9.0 g/dL, white blood cell counts ≥3000/mm^3^ and neutrophil counts ≥2000/mm^3^); (7) Adequate liver function (bilirubin ≤1.5 mg/dL, aspartate aminotransferase (AST) and alanine aminotransferase (ALT) ≤2.5 times the upper limit of normal range (ULN) or ≤5-times the ULN if liver metastasis is present); (8) Adequate kidney function (creatinine levels ≤1.5 mg/dL); (9) Life expectancy of at least 12 weeks. Patients were excluded from the study if they had a history of serious drug hypersensitivity; concurrent treatment with atazanavir sulphate; persistent ≥ grade 2 adverse reactions due to prior therapy except for alopecia and anaemia; anticancer treatment within the 3 weeks and/or extensive radiation therapy within the 6 weeks prior to the start of study treatment; other concurrent cancer; brain metastasis; or if they were pregnant or breast-feeding women. During the study, granulocyte colony stimulating factor (G-CSF) was allowed except for prophylactic use.

The study was conducted in accordance with the Declaration of Helsinki and the Japanese Good Clinical Practice guideline. Written informed consent was obtained from all patients. This study was approved by the Ethics Committees of the participating institutions (National Cancer Center Hospital East, Shizuoka Cancer Center, Kitasato University East Hospital and Showa University Northern Yokohama Hospital). (JapicCTI-No.: JapicCTI-132099).

### Treatment

**Fig. 1:**
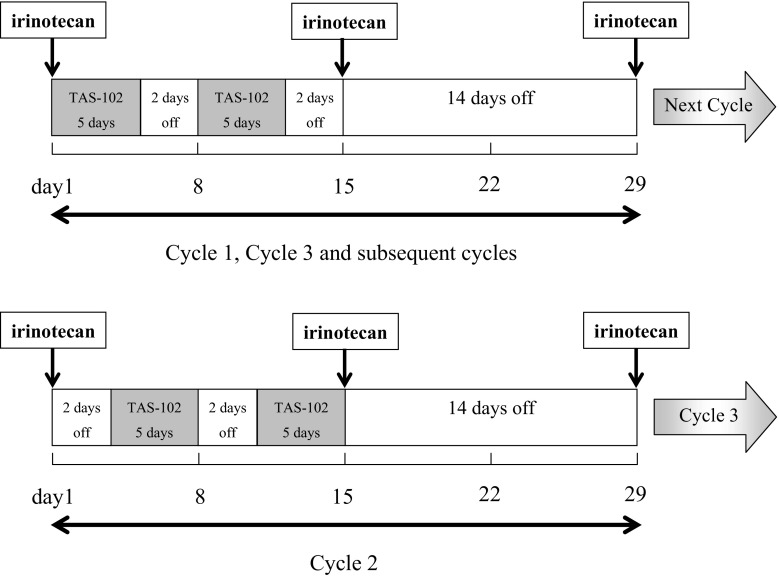
Study drug dosing schedule (Legend: During Cycle 2, to allow for pharmacokinetic assessment of irinotecan alone, TAS-102 was administered twice daily, on Days 3–7 and Days 10–14 of the 28-day treatment cycle.)

As depicted in Fig. 1, during all cycles, irinotecan was administered by intravenous infusion over at least 90 min on Days 1 and 15 in a 28-day schedule. The initial irinotecan dose was 150 mg/m^2^. TAS-102 was administered twice daily, after the morning and evening meal, for 5 days a week with 2 days rest for 2 weeks, followed by a 14-day rest (1 treatment cycle). This treatment cycle was repeated every 4 weeks. The dose of TAS-102 was set at one of four dose levels (Level 0: 40 mg/m^2^/day; Level 1: 50 mg/m^2^/day; Level 2: 60 mg/m^2^/day; and Level 3: 70 mg/m^2^/day), starting at Level 1. In Cycle 2, to assess the pharmacokinetics of irinotecan alone, irinotecan was administered as described above, and TAS-102 was administered on Days 3–7 and Days 10–14 of the 28-day treatment cycle.

Dose reductions of TAS-102 and irinotecan due to toxicities were not allowed unless DLT was observed during the Cycle 1, and thereafter permitted according to the pre-specified criteria. Study treatment was continued until investigator-judged progressive disease, adverse event(s) requiring discontinuation, a treatment-free period of >30 consecutive days, withdraw of consent to continue the protocol treatment.

Actual dose intensity (mg/m^2^/weeks) of TAS-102 and irinotecan was defined as cumulative dose (mg/m^2^) divided by the number of weeks from initial treatment to discontinuation. Relative dose intensity (%) was calculated based on the initial planned dose.

### Toxicity assessment and dose-escalation procedure

Examination of patient’s condition and laboratory tests were repeated weekly. Adverse events were graded according to the National Cancer Institute’s Common Terminology Criteria for Adverse Events (CTCAE), version 3.0.

The following adverse drug reactions were defined as the DLT: ≥grade 3 non-haematological toxicities (excluding nausea, vomiting and diarrhoea); ≥grade 3 nausea, vomiting or diarrhoea showing no improvement even after supportive treatment; ≥grade 3 febrile neutropenia; grade 4 neutropenia persisting for ≥5 days; grade 4 thrombocytopenia; grade 4 other non-haematological toxicities; or delay of starting Cycle 2 longer than 14 days due to adverse drug reaction.

TAS-102 was administered to 3 patients at each dose level. If 1 of the 3 patients experienced a DLT, 3 more patients were enrolled at the same dose level. The Maximum tolerated dose (MTD) was defined as the dose level at which 2 or more of 3 patients, or at least 2 of 4 to 6 patients, had DLTs during Cycle 1. The RD was defined as one dose level lower than the MTD.

### Pharmacokinetic assessment

To see the PK profiles of irinotecan when dosed alone and in combination with TAS-102, blood samples were collected before dosing and at 15, 30 min, 1, 2, 2.5, 4, 6, 8, 10, 24 and 48 h after initial dosing on day 1 in Cycles 1 and 2. In addition, blood collections were carried out immediately before the end of infusion on day 1 in the both Cycles.

The plasma concentrations of FTD, its inactive metabolite trifluorothymine (FTY), and TPI were measured using validated liquid chromatography-tandem mass spectrometry. The plasma concentrations of irinotecan and SN-38 were measured using validated high performance liquid chromatography. Blood samples at 24 and 48 h were excluded from analysis for FTD, FTY and TPI. Concentrations of TPI and irinotecan were obtained as those of hydrochloride and hydrochloride hydrate, respectively.

For FTD, FTY and TPI, the following were calculated by non-compartmental analysis using WinNonlin (Pharsight Corporation): maximum plasma concentration (C_max_), time to maximum plasma concentration (T_max_), area under the plasma concentration versus time curve (AUC; measured as AUC_0-t_ and AUC_0-inf_) and elimination half-life (t_½_). Oral clearance (CL/F) and apparent volume of distribution (Vd/F) were also calculated except for FTY. For irinotecan and SN-38, the following were calculated by non-compartmental analysis: C_max_, AUC_0-t_, AUC_0-inf_, t_½_, total body clearance (CL_tot_) and Vd.

### Tumor assessments

Imaging examination for tumor assessment was performed within 14 days prior to treatment, and repeated every 4–6 weeks, and at treatment completion/discontinuation. The antitumor efficacy (best overall response), disease control rate (DCR), time to treatment failure (TTF) and progression-free survival (PFS) were assessed by the investigator. The antitumor efficacy was evaluated according to RECIST version 1.0.

### *KRAS* status and UGT1A1

Tumor tissue samples, at least 5 pieces of formalin-fixed paraffin-embedded specimens, were obtained for analysis of the presence or absence of *KRAS* mutation (codons 12 and 13) by real-time polymerase chain reaction [PCR; Scorpions-ARMS, TheraScreen *KRAS* (DxS Ltd. kit)] in a central laboratory.

The potential relationship between adverse reactions with the UGT1A1 polymorphism (determined for *6,*27 and *28; a predictor of irinotecan toxicity) was also examined. DNA was extracted from blood samples and analysed for UGT1A1 polymorphism using the Invader method (UGT1A1 polymorphism assessment kit, Third Wave Japan, Inc.) in a central laboratory.

### Sample size and statistical analysis

The number of patients in each cohort was based on a standard 3 plus 3 design for dose-escalation studies. A maximum of 24 patients were planned to be enrolled in the study. The primary analysis (and all efficacy analyses discussed herein) included the full analysis set (FAS), which was defined as eligible patients treated with TAS-102 and irinotecan at least once who could be evaluated for DLT. The safety analyses included all treated patients.

The effect of TAS-102 on the PK of irinotecan and SN-38 was assessed by comparing PK parameters, C_max_ and AUC, derived from patients received the same dose between initial administration in Cycle 1 (combination use) and Cycle 2 (irinotecan alone). The effect of irinotecan on the PK of TAS-102 was assessed by comparing the PK parameters of FTD, FTY and TPI in each dose level of Cycle 1 with those of patients received TAS-102 alone at the same dose level in Japanese phase I study [8]. PK parameters were converted to common logarithms and then assessed using the paired student’s *t*-test for irinotecan and SN-38 and using the unpaired student’s *t*-test for FTD, FTY and TPI (significance level for each test: 5 %). SAS for Windows (SAS Institute Inc.) was used as the statistical analysis software.

## Results

Twelve patients were screened for this study, and 10 patients were enrolled and treated: 7 at Level 1 and 3 at Level 2. Of the 10 eligible patients, 9 (6 patients at Level 1 and 3 patients at Level 2) were included in the FAS population because one patient at Level 1 withdrew consent before irinotecan administration on day 15 in Cycle 1 and no follow-up data was available, making it difficult to evaluate DLTs, safety or efficacy.

### Patient characteristics

Table 1 shows the background of the all ten patients enrolled to this study. The majority of patients were male, with an age range of 31 to 72 years. Eight patients had a PS of 0, and 2 patients had a PS of 1. Six patients had recurrent disease, and 4 patients had unresectable disease. All patients had resection of primary lesions and metastatic lesions (liver, 7 patients; abdominal lymph node, 4 patients; lung and others, 3 patients each; and thoracic lymph node, 2 patients). Eight patients had history of bevacizumab use and no patient had received anti-epidermal growth factor receptor monoclonal antibody. *KRAS* status was available from all patients, in whom *KRAS* mutation was present in 4 patients. Heterozygotes for the UGT1A1 polymorphisms *6, *27 and *28 were detected in 1, 0 and 4 patients, respectively, while the other patients had the wild-type (*1). None of the patients had homozygous or double heterozygous variations.

**Table 1:** Patient Characteristics

	Level 1 (*N* = 7)	Level 2 (*N* = 3)	Total (*N* = 10)
	N (%)	N (%)	N (%)
Median age (range), years	57.0 (31–72)	71.0 (48–72)	59.0 (31–72)
Gender			
Male	5 (71.4)	2 (66.7)	7 (70.0)
Female	2 (28.6)	1 (33.3)	3 (30.0)
ECOG Performance Status			
0	6 (85.7)	2 (66.7)	8 (80.0)
1	1 (14.3)	1 (33.3)	2 (20.0)
Cancer Diagnosis			
Recurrent	3 (42.9)	3 (100.0)	6 (60.0)
Unresectable	4 (57.1)	0 (0.0)	4 (40.0)
Primary Lesion			
Colon	5 (71.4)	1 (33.3)	6 (60.0)
Rectum	2 (28.6)	2 (66.7)	4 (40.0)
Histological Type			
Well-differentiated	1 (14.3)	2 (66.7)	3 (30.0)
Moderately differentiated	5 (71.4)	1 (33.3)	6 (60.0)
Poorly differentiated	1 (14.3)	0 (0.0)	1 (10.0)
Prior Therapies			
Surgery	7 (100.0)	3 (100.0)	10 (100.0)
Adjuvant chemotherapy	3 (42.9)	3 (100.0)	6 (60.0)
1st line chemotherapy	7 (100.0)	3 (100.0)	10 (100.0)
Bevacizumab	6 (85.7)	2 (66.7)	8 (80.0)
Cetuximab	0 (0.0)	0 (0.0)	0 (0.0)
*KRAS* status			
wild-type	4 (57.1)	2 (66.7)	6 (60.0)
mutant	3 (42.9)	1 (33.3)	4 (40.0)
UGT1A1 polymorphism^a^			
wild-type	3 (42.9)	2 (66.7)	5 (50.0)
UGT1A1*6	0 (0.0)	1 (33.3)	1 (10.0)
UGT1A1*27	0 (0.0)	0 (0.0)	0 (0.0)
UGT1A1*28	4 (57.1)	0 (0.0)	4 (40.0)

Analysis set is all treated patients

Abbreviations: *ECOG* Eastern Cooperative Oncology Group

^a^None of the patients had homozygous or double heterozygous variations

### TAS-102 and irinotecan administration

In the 10 treated patients with at least one administration of the protocol treatment, the median total duration of TAS-102 dosing was 26.3 days at Level 1 and 113.0 days at Level 2. The median total number of irinotecan dosing was 4.5 times at Level 1 and 12.0 times at Level 2. The median duration of treatment was 69.0 days at Level 1 and 401.0 days at Level 2, and the largest number of cycles of the combination therapy was 6 at Level 1 and 29 at Level 2.

In the 9 patients of the FAS, dose reduction of TAS-102 was required in 1 of 6 patients at Level 1 and 2 of 3 patients at Level 2, and dose reduction of irinotecan (from 150 mg/m^2^ to 120 mg/m^2^) was done in 1 of 6 patients at Level 1 and 3 of 3 patients at Level 2. Interruption of TAS-102 dosing was required in 3 of 6 patients at Level 1 and 2 of 3 patients at Level 2, and the second administration of irinotecan in the first Cycle was delayed in 5 of 6 patients at Level 1 and 3 of 3 patients at Level 2. The median relative dose intensity of TAS-102 was 84.25 % at Level 1 and 68.28 % at Level 2, and those of irinotecan were 68.86 % at Level 1 and 32.59 % at Level 2. The relative dose intensity was ≥80 % in 4 of 6 patients at Level 1 and 1 of 3 patients at Level 2 for TAS-102, and in 1 of 6 patients at Level 1 and none of 3 patients at Level 2 for irinotecan.

### DLTs and RD

A total of 5 DLT events occurred in 3 patients; 2 DLTs in 1 patient at Level 1 and 3 DLTs in 2 patients at Level 2. One patient at Level 1 experienced Grade 4 neutropenia persisting for ≥5 days and Grade 3 febrile neutropenia in Cycle 1 on day 22. Although neutrophil count recovered on day 33 without G-CSF this patients discontinued the study due to disease progression. This patient had previously received radiofrequency ablation, heterozygote for the UGT1A1 polymorphisms *28 were detected. One patient at Level 2 experienced Grade 4 neutropenia persisting for ≥5 days in Cycle 1 on day 10 and Grade 3 febrile neutropenia in Cycle 1 on day 15 resulting in skip of second administration of irinotecan. Neutrophil count recovered on day 28 by an antibiotic and started a Cycle 2 with dose reduction of irinotecan. No other DLTs occurred after that, this patient continued a study treatment to Cycle 12 (TTF: 401 days). Heterozygotes for the UGT1A1 polymorphisms were not detected. The other patient at Level 2 experienced Grade 3 febrile neutropenia in Cycle 1 on day 19. Neutrophil count recovered on day 26 by G-CSF. This patient discontinued the study due to disease progression at Cycle 2. Heterozygote for the UGT1A1 polymorphisms *6 was detected

The MTD of this combination therapy was estimated to be 60 mg/m^2^/day (Level 2) TAS-102 with 150 mg/m^2^/day irinotecan. The RD was determined as 50 mg/m^2^/day TAS-102 (Level 1) with 150 mg/m^2^/day irinotecan.

### Safety and tolerability

All 10 treated patients who received TAS-102 and irinotecan experienced at least one treatment-related adverse event. The common treatment-related adverse events are summarized in Table 2. In this study, the most common treatment-related adverse events were bone marrow suppression, diarrhoea, nausea, malaise, decreased appetite and alopecia. While all symptomatic treatment-related adverse event, including gastrointestinal symptoms except for diarrhoea and nausea, were grade 2 or lower, grade 3 or higher treatment-related adverse event were related to bone marrow suppression. Two cases of grade 4 neutropenia occurred in 2 patients at Level 1, and 12 episodes of grade 4 neutropenia occurred in 3 patients at Level 2. The bone marrow suppression in all patients was reversible.

**Table 2:** Most common treatment-related adverse events (all cycles)

	Level 1 (*N* = 7)		Level 2 (*N* = 3)		Total (*N* = 10)	
	All grades	Grade 3/4	All grades	Grade 3/4	All grades	Grade 3/4
	N (%)	N (%)	N (%)	N (%)	N (%)	N (%)
Haematological toxicities						
Neutrophil count decreased	7 (100.0)	7 (100.0)	3 (100.0)	3 (100.0)	10 (100.0)	10 (100.0)
White blood cell count decreased	7 (100.0)	4 (57.1)	3 (100.0)	3 (100.0)	10 (100.0)	7 (70.0)
Lymphocyte count decreased	6 (85.7)	2 (28.6)	2 (66.7)	0 (0.0)	8 (80.0)	2 (20.0)
Haemoglobin decreased	6 (85.7)	0 (0.0)	2 (66.7)	2 (66.7)	8 (80.0)	2 (20.0)
Haematocrit decreased	6 (85.7)	0 (0.0)	2 (66.7)	0 (0.0)	8 (80.0)	0 (0.0)
Red blood cell count decreased	6 (85.7)	0 (0.0)	2 (66.7)	0 (0.0)	8 (80.0)	0 (0.0)
Platelet count decreased	4 (57.1)	0 (0.0)	3 (100.0)	1 (33.3)	7 (70.0)	1 (10.0)
Febrile neutropenia	1 (14.3)	1 (14.3)	2 (66.7)	2 (66.7)	3 (30.0)	3 (30.0)
Anaemia	2 (28.6)	2 (28.6)	1 (33.3)	0 (0.0)	3 (30.0)	2 (20.0)
Blood albumin decreased	2 (28.6)	0 (0.0)	0 (0.0)	0 (0.0)	2 (20.0)	0 (0.0)
Protein total decreased	2 (28.6)	0 (0.0)	0 (0.0)	0 (0.0)	2 (20.0)	0 (0.0)
Non-haematological toxicities						
Decreased appetite	6 (85.7)	0 (0.0)	3 (100.0)	0 (0.0)	9 (90.0)	0 (0.0)
Diarrhoea	6 (85.7)	0 (0.0)	2 (66.7)	0 (0.0)	8 (80.0)	0 (0.0)
Malaise	5 (71.4)	0 (0.0)	3 (100.0)	0 (0.0)	8 (80.0)	0 (0.0)
Nausea	5 (71.4)	0 (0.0)	2 (66.7)	0 (0.0)	7 (70.0)	0 (0.0)
Alopecia	4 (57.1)	0 (0.0)	2 (66.7)	0 (0.0)	6 (60.0)	0 (0.0)
Vomiting	2 (28.6)	0 (0.0)	1 (33.3)	0 (0.0)	3 (30.0)	0 (0.0)
Abdominal pain	2 (28.6)	0 (0.0)	1 (33.3)	0 (0.0)	3 (30.0)	0 (0.0)
Stomatitis	2 (28.6)	0 (0.0)	0 (0.0)	0 (0.0)	2 (20.0)	0 (0.0)
Constipation	1 (14.3)	0 (0.0)	1 (33.3)	0 (0.0)	2 (20.0)	0 (0.0)

Adverse events coded using MedDRA (version 15.1)

None of the patients died within 90 days from initiating treatment or within 30 days after completion (discontinuation) of treatment. No patients discontinued the study due to treatment-related adverse event. Two serious adverse events (ascites and blood bilirubin increased) occurred in 1 patient at Level 1, and 2 serious treatment-related adverse events (diarrhoea and febrile neutropenia) occurred in 1 patient at Level 2. The diarrhoea and febrile neutropenia resolved with appropriate treatment.

### Pharmacokinetics

The effect of TAS-102 on the PK of irinotecan was assessed in the 5 patients; four of the 7 patients at Level 1 and 1 of the 3 patients at Level 2 who received same dosage of irinotecan during the first and the second Cycle because two patients at Level 1 discontinued the study treatment during the first Cycle due to progressive disease or consent withdrawal, and 3 patients (1 at Level 1, 2 at Level 2) had dose reduction at the start of the second Cycle. No significant differences were observed in the PK parameters, such as C_max_, AUC_0-t_ and AUC_0-inf_ of irinotecan and SN-38 between irinotecan alone and combined administration with TAS-102 (Table 3). The effect of irinotecan on the PK of TAS-102 was assessed in the 7 patients at Level 1 and 3 patients at Level 2. Nor were any significant interaction by irinotecan on PK parameters of FTD, FTY and TPI compared to Japanese phase I study of TAS-102 monotherapy (Table 4).

**Table 3:** Pharmacokinetic Parameters of irinotecan and SN-38 at Level 1 and Level 2 (mean ± SD)

Treatment		Irinotecan Plus TAS-102	Irinotecan Alone	Irinotecan Plus TAS-102	Irinotecan Alone
Compound		irinotecan^c^		SN-38	
Level 1	n	7	4	7	4
	C_max_ (ng/mL)	2230 ± 470	1980 ± 170	36.9 ± 23.2	33.3 ± 14.0
	AUC_0-t_ (hr.ng/mL)	13,000 ± 4700	10,200 ± 1700	327 ± 194	233 ± 82
	AUC_0-inf_ (hr.ng/mL)	13,300 ± 4900	10,300 ± 1800	426 ± 277	278 ± 86
	t_½_ (hr)	9.54 ± 1.04	8.52 ± 0.94	22.0 ± 6.2	19.4 ± 5.2
	CL_tot_ (L/hr/m^2^)^a^	12.7 ± 4.5	14.9 ± 2.6	NR	NR
	Vd (L/m^2^) ^b^	170 ± 47	181 ± 19	NR	NR
Level 2	n	3	1	3	1
	C_max_ (ng/mL)	2150 ± 150	1660	51.9 ± 32.1	37.3
	AUC_0-t_ (hr.ng/mL)	14,100 ± 4600	9360	493 ± 258	366.0
	AUC_0-inf_ (hr.ng/mL)	14,400 ± 4700	9480	619 ± 318	412
	t_½_ (hr)	8.94 ± 0.75	8.6	24.1 ± 1.7	17.1
	CL_tot_ (L/hr/m^2^)^a^	11.1 ± 3.1	15.8	NR	NR
	Vd (L/m^2^)^b^	144 ± 43	196	NR	NR

Abbreviations: *NR* not reported, *SD* standard deviation; and SN‑38 = 7‑ethyl-10‑hydroxycamptothecin

^a^CLtot (L/hr/m^2^) for irinotecan

^b^Vd (L/m^2^) for irinotecan

^c^Concentrations of irinotecan was obtained as those of hydrochloride hydrate

**Table 4:** Pharmacokinetic Parameters of FTD, FTY, and TPI at Level 1 and Level 2 (mean ± SD)

PK Parameters		Level 1 (50 mg/m^2^/day)		Level 2 (60 mg/m^2^/day)	
		Irinotecan Plus TAS-102 (*N* = 7)	TAS-102 Alone^a^ (*N* = 3)	Irinotecan Plus TAS-102 (*N* = 3)	TAS-102 Alone^a^ (*N* = 3)
FTD	C_max_ (ng/mL)	2740 ± 770	2450 ± 1021	3290 ± 1380	3677 ± 1459
	T_max_ (hr)	1.18 ± 0.85	1.5 ± 0.9	1.33 ± 0.76	1.2 ± 0.8
	AUC_0-t_ (hr.ng/mL)	5277 ± 1673	NR	6672 ± 1383	NR
	AUC_0-inf_ (hr.ng/mL)	5322 ± 1684	4297 ± 1387	6823 ± 1508	8435 ± 1645
	t_½_ (hr)	1.67 ± 0.31	1.49 ± 0.59	2.10 ± 0.36	1.88 ± 0.73
	CL/F (L/hr/kg)	0.143 ± 0.088	0.178 ± 0.055	0.129 ± 0.028	0.103 ± 0.014
	Vd/F (L/kg)	0.361 ± 0.267	0.384 ± 0.175	0.385 ± 0.070	0.273 ± 0.089
FTY	C_max_ (ng/mL)	614 ± 94	645 ± 23	856 ± 274	753 ± 293
	T_max_ (hr)	1.64 ± 0.69	1.5 ± 0.9	1.83 ± 0.76	1.5 ± 0.9
	AUC_0-t_ (hr.ng/mL)	1859 ± 187	NR	2867 ± 198	NR
	AUC_0-inf_ (hr.ng/mL)	1900 ± 192	1915 ± 327	2958 ± 182	2710 ± 559
	t_½_ (hr)	1.91 ± 0.41	1.18 ± 0.18	1.77 ± 0.14	1.62 ± 0.32
TPI^b^	C_max_ (ng/mL)	82.7 ± 30.0	54.2 ± 28.5	89.5 ± 11.4	136.1 ± 77.5
	T_max_ (hr)	1.86 ± 0.56	1.7 ± 0.6	2.50 ± 0.00	2.7 ± 1.2
	AUC_0-t_ (hr.ng/mL)	256 ± 100	NR	382 ± 20	NR
	AUC_0-inf_ (hr.ng/mL)	265 ± 103	222 ± 79	414 ± 30	542 ± 360
	t_½_ (hr)	2.05 ± 0.30	1.78 ± 0.27	2.17 ± 1.13	1.66 ± 0.37
	CL/F (L/hr/kg)	1.40 ± 0.83	1.66 ± 0.56	0.978 ± 0.072	0.91 ± 0.40
	Vd/F (L/kg)	4.29 ± 2.88	4.31 ± 1.85	2.99 ± 1.33	2.06 ± 0.62

Abbreviations: *FTD* trifluridine (α,α,α‑trifluorothymidine), *FTY* trifluorothymine, *NR* not reported, *SD* standard deviation; SN‑38 = 7‑ethyl-10‑hydroxycamptothecin; and TPI = tipiracil hydrochloride (thymidine phosphorylase inhibitor)

^a^Phase I study of TAS-102 monotherapy. TAS-102 was administered twice daily, after the morning and the evening meal, for 5 days a week with 2 days rest for 2 weeks, followed by a 14-day rest

^b^Concentrations of TPI was obtained as those of hydrochloride

### Efficacy

**Fig. 2:**
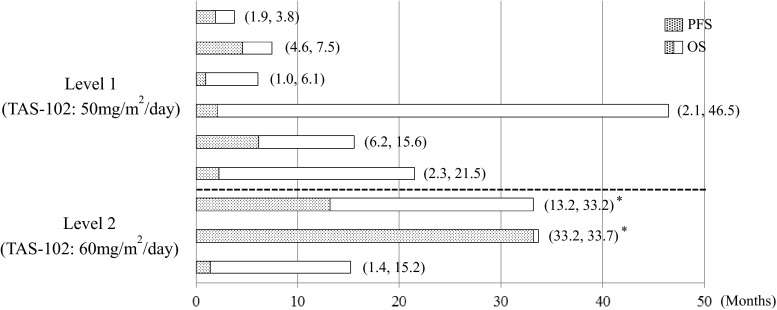
Individual progression-free survival and overall survival (PFS, OS) (Legend: *Survival follow up was terminated because of study completion.)

As shown in Table 5, the response rate was 16.7 % at Level 1, 33.3 % at Level 2 and 22.2 % overall (duration of response were 112 and 799 days). The disease control rate was 50.0 % at Level 1, 66.7 % at Level 2, and 55.6 % overall. The median PFS was 2.2 months (95 % confidence interval [CI], 1.9–4.6 months) at Level 1 and 13.2 months (95 % CI, 1.4–33.7 months) at Level 2. The median TTF was 2.2 months (95 % CI, 1.9–4.6 months) at Level 1 and 13.2 months (95 % CI, 1.4–33.2 months) at Level 2. The median OS was 11.6 months (95 % CI, 6.1–21.5 months) at Level 1 and was not reached (95 % CI, 15.2 months–not reached) at Level 2 with median follow-up time 33.7 months (range, 33.2–46.5 months). Individual OS and PFS were shown in Fig. 2.

**Table 5:** Efficacy summary

	Level 1 (*N* = 6)	Level 2 (*N* = 3)	Total (*N* = 9)
	N (%)	N (%)	N (%)
CR	0 (0.0)	0 (0.0)	0 (0.0)
PR	1 (16.7)	1 (33.3)	2 (22.2)
SD	2 (33.3)	1 (33.3)	3 (33.3)
PD	3 (50.0)	1 (33.3)	4 (44.4)
NE	0 (0.0)	0 (0.0)	0 (0.0)
Response Rate (CR + PR)	1 (16.7)	1 (33.3)	2 (22.2)
[95 % CI]	[0.4–64.1]	[0.8–90.6]	[2.8–60.0]
Disease Control Rate (CR + PR + SD)	3 (50.0)	2 (66.7)	5 (55.6)
[95 % CI]	[11.8–88.2]	[9.4–99.2]	[21.2–86.3]
OS (months)	11.6 (6.1–21.5)	NR (15.2-NR)	15.6 (7.5-NR)
PFS (months)	2.2 (1.9–4.6)	13.2 (1.4–33.7)	2.3 (1.9–6.2)
TTF (months)	2.2 (1.9–4.6)	13.2 (1.4–33.2)	2.3 (1.9–6.2)

Abbreviations: *CI* confidence interval, *CR* complete response, *NE* not evaluable, *NR* not reached, *PD* progressive disease, *PR* partial response; and *SD* stable disease

### *KRAS* status and UGT1A1

The *KRAS* mutation was detected in 3 of 9 patients in the FAS population. The response rate in patients with and without the *KRAS* mutation was 0 and 33.3 %, and the disease control rate was 33.3 and 66.7 %. The median PFS in the patients with and without the *KRAS* mutation was 1.4 months (95 % CI, 1.0–4.6 months) and 4.3 months (95 % CI, 2.1–13.2 months), respectively. The median TTF in the patients with and without the *KRAS* mutation was 1.4 months (95 % CI, 1.0–4.6 months) and 4.3 months (95 % CI, 2.1–13.2 months), respectively. The median OS was not reached (95 % CI, 15.6 months–not reached) in patients with wild-type *KRAS* and 7.5 months (95 % CI, 6.1–15.2 months) in patients with the *KRAS* mutation.

Variant polymorphisms for the UGT1A1 at *6, *27 and *28 were detected in 1, 0 and 4 patients out of all the enrolled 10 patients, respectively. One of nine patients with wild-type UGT1A1*6 experienced grade 4 thrombocytopenia, and 1 patient with heterozygous UGT1A1*6 experienced grade 4 leukopenia and neutropenia. Among the 6 patients with wild-type UGT1A1*28, 1 and 4 patients experienced grade 4 leukopenia and neutropenia, and another patient experienced grade 4 thrombocytopenia, while among the 4 patients with heterozygous UGT1A1*28, 1 patient experienced grade 4 neutropenia.

Variant polymorphisms were detected in 5 patients who experienced a DLT. One patient with heterozygous UGT1A1*6 experienced grade 3 febrile neutropenia, and 2 patients with heterozygous UGT1A1*28 experienced grade 3 febrile neutropenia and grade 4 neutropenia.

## Discussion

The primary objective of this study was to determine the RD and schedule for future clinical studies, and to evaluate the safety of the combination of TAS-102 plus irinotecan in patients with metastatic colorectal cancer. The dose of irinotecan used in combination with TAS-102 in this study was 150 mg/m^2^ (every 2 weeks), which is the Japanese approved dose of irinotecan alone.

MTD was estimated to be 60 mg/m^2^/day TAS-102 with 150 mg/m^2^/day irinotecan, and RD was determined one dose level below of MTD. The most common treatment-related adverse event was bone marrow suppression, such as neutropenia, leukopenia and lymphopenia, the frequency and grade in this study were higher to that in TAS-102 monotherapy or other irinotecan containing regimen [9, 12–14]. Especially, Grade 3 or higher neutropenia occurred in all patients and 3 of them were associated with febrile neutropenia. All of 3 patients experienced febrile neutropenia in Cycle 1 with the range of Day 15 to Day 22 and rapidly improved by dose reductions, temporary interruptions or administration of antibiotics or G-CSF. All toxicities were reversible and resolved by appropriate measures.

The combination of TAS-102 plus irinotecan showed favourable tumor response in patients who were refractory to 5-FU and oxaliplatin. The response rate (22.2 %) was comparable to other conventional chemotherapies [12–14]. The difference of mechanism of action between TAS-102 and 5-FU is clinically important because the great portion of metastatic colorectal cancer patients receive 5-FU containing regimen sequentially (e.g., FOLFOX followed by FOLFIRI) even if refractory to 5-FU. Antitumor efficacy of TAS-102 to 5-FU resistance was confirmed not only preclinical studies but also a phase III study (RECOURSE), included approximately 50 % of patients who were received 5-FU in their most recent treatment and had disease progression [11, 15]. Therefore it is meaningful to continue to develop this combination regimen in earlier treatment line.

There are limitation and issue to be resolved in this study. First, sample size was extremely limited because of early onset of DLTs, not allowed statistical adjustments and exploratory analysis including *KRAS* status and UGT1A1. In addition, we were not able to robustly evaluating the PK analysis. Although there might be no mutually significant effect on the PK of each drug, further investigation with large sample size is needed. Second, the relative dose intensity of irinotecan, especially at Level 2, was lower than that of other irinotecan containing regimens [12, 13]. Neutrophil count nadir was occurred around Day 22 in each cycle, however closed with nadir at Day 15 (data not shown). Given the fact that the toxicity of TAS-102 monotherapy tended to be observed in Day 21 [8], it was speculated that the augmentation and earlier appearance of bone marrow suppression by overlapping toxicity of both drugs interfered the second administration of irinotecan.

For future plan, explorations of the refined regimen to improve dose intensity are necessary. It was reported that the antitumor effect of TAS-102 was dose dependent (30–70 mg/m^2^/day) [8]. A preclinical study also revealed that the antitumor activity of TAS-102 had a positive correlation with the amount of FTD incorporated into DNA in various tumor cell lines [16]. Actually the efficacy at Level 2, although small sample size, was better than Level 1 in this study. Therefore, it is well suited to adjust the treatment schedule without reducing dose of each drug. Additionally, investigation with a molecular target is important in light of recent clinical evidence [17, 18]. A phase I clinical study using a new treatment schedule with a molecular target is currently in progress (ClinicalTrials.gov Identifier: NCT01916447).

In summary, the RD was determined to be 50 mg/m^2^/day of TAS-102 combined with 150 mg/m^2^ of irinotecan, although further investigation to explore optimal regimen is warranted.
